# (*E*)-*N*′-Hy­droxy-1,3-diphenyl-4,5-di­hydro-1*H*-pyrazole-5-carboximidamide

**DOI:** 10.1107/S1600536812019630

**Published:** 2012-05-05

**Authors:** Chandra, N. Srikantamurthy, K. B. Umesha, S. Jeyaseelan, M. Mahendra

**Affiliations:** a Department of Studies in Physics, Manasagangotri, University of Mysore, Mysore 570 006, India; b Department of Chemistry, Yuvaraja’s College, University of Mysore, Mysore 570 005, India; c Department of Physics, St Philomena’s College, Mysore, India

## Abstract

In the mol­ecule of the title compound, C_16_H_16_N_4_O, the pyrazole ring makes dihedral angles of 8.52 (13) and 9.26 (12)° with the phenyl rings. The dihedral angle between the benzene rings is 1.86 (13)°. In the crystal, mol­ecules are linked into centrosymmetric dimers *via* pairs of O—H⋯N hydrogen bonds. Weak N—H⋯N inter­actions connect the dimers into a chain along the [100] direction. The pyrazole ring adopts a highly flattened envelope conformation.

## Related literature

For the biological activity of pyrazoles, see: Da Sliva *et al.* (2010 ▶); Farag *et al.* (2010 ▶); Khode *et al.* (2009 ▶); Boschi *et al.* (2011 ▶); Ghorab *et al.* (2010 ▶); Husain *et al.* (2008 ▶); Taj *et al.* (2011 ▶); Mikhaylichenko *et al.* (2009 ▶). For bond-length data, see: Allen *et al.* (1987 ▶). For puckering parameters, see: Cremer & Pople (1975 ▶). For a related structure, see: Fun *et al.* (2011 ▶).
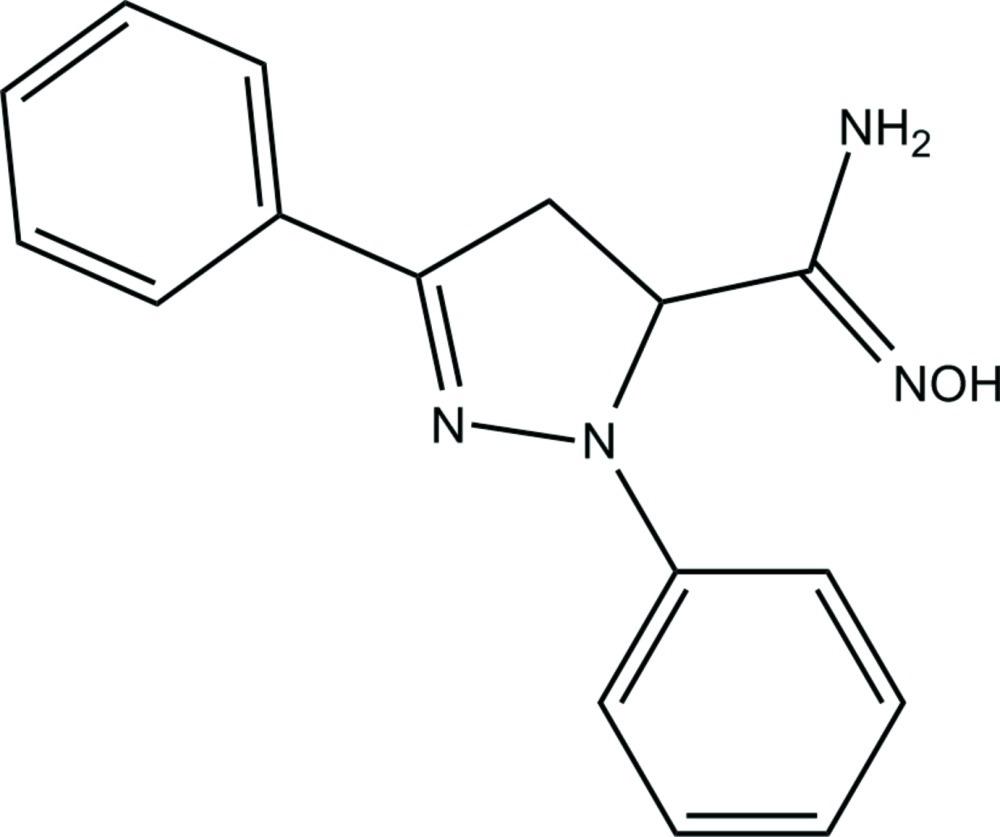


## Experimental

### 

#### Crystal data

C_16_H_16_N_4_O
*M* *_r_* = 280.33
Triclinic, 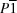

*a* = 7.845 (11) Å
*b* = 8.940 (12) Å
*c* = 11.116 (15) Å
α = 99.50 (2)°
β = 99.76 (2)°
γ = 106.70 (2)°
*V* = 716.8 (17) Å^3^
*Z* = 2
Mo *K*α radiation
μ = 0.09 mm^−1^
*T* = 293 K
0.30 × 0.22 × 0.15 mm

#### Data collection

Bruker APEXII CCD area-detector diffractometer
6110 measured reflections
2480 independent reflections
1912 reflections with *I* > 2σ(*I*)
*R*_int_ = 0.031

#### Refinement

*R*[*F* ^2^ > 2σ(*F* ^2^)] = 0.053
*wR*(*F* ^2^) = 0.172
*S* = 1.09
2480 reflections
192 parameters
H-atom parameters constrained
Δρ_max_ = 0.22 e Å^−3^
Δρ_min_ = −0.29 e Å^−3^

### 

Data collection: *APEX2* (Bruker, 2009 ▶); cell refinement: *SAINT* (Bruker, 2009 ▶); data reduction: *SAINT*; program(s) used to solve structure: *SHELXS97* (Sheldrick, 2008 ▶); program(s) used to refine structure: *SHELXL97* (Sheldrick, 2008 ▶); molecular graphics: *PLATON* (Spek, 2009 ▶); software used to prepare material for publication: *SHELXL97*.

## Supplementary Material

Crystal structure: contains datablock(s) global, I. DOI: 10.1107/S1600536812019630/ds2183sup1.cif

Structure factors: contains datablock(s) I. DOI: 10.1107/S1600536812019630/ds2183Isup2.hkl

Supplementary material file. DOI: 10.1107/S1600536812019630/ds2183Isup3.cml

Additional supplementary materials:  crystallographic information; 3D view; checkCIF report

## Figures and Tables

**Table 1:** Hydrogen-bond geometry (Å, °)

*D*—H⋯*A*	*D*—H	H⋯*A*	*D*⋯*A*	*D*—H⋯*A*
N7—H7*B*⋯N3^i^	0.86	2.62	3.449 (5)	164
O9—H9⋯N8^ii^	0.82	2.12	2.829 (5)	145

Symmetry codes: (i) 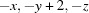; (ii) 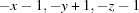.

**Fig. 1.**
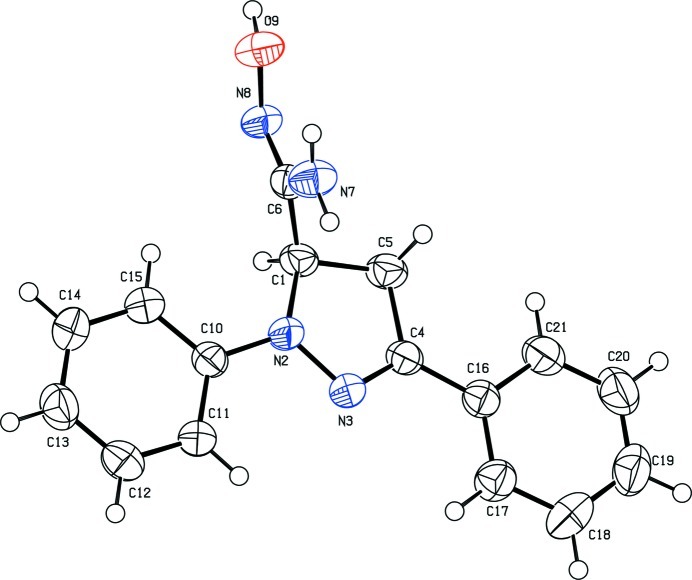
Perspective diagram of the molecule with 50% probability displacement ellipsoids.

**Fig. 2.**
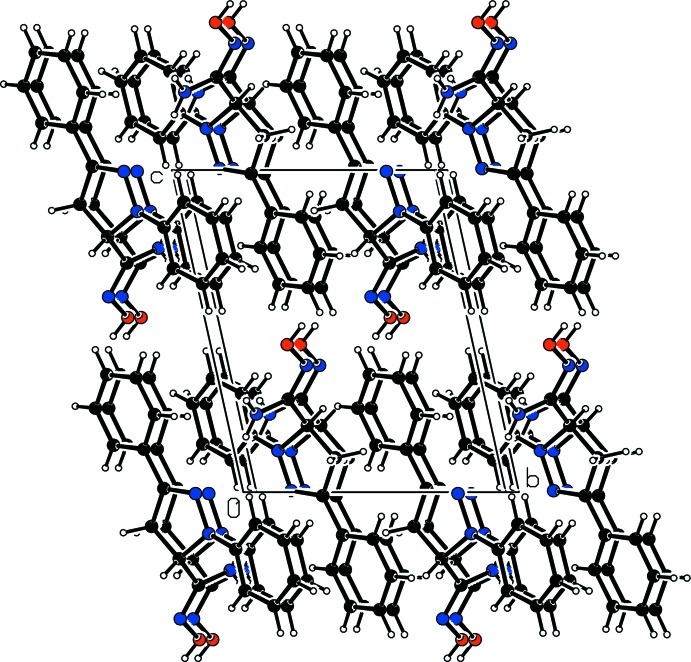
Packing diagram of the molecule viewed down the 'a' axis. The dotted lines represents the hydrogen bonds.

## supplementary crystallographic information

### Comment

Pyrazole and its derivatives are a class of five-membered heterocyclic structure with two adjacent nitrogen atoms. These derivatives have drawn more attention in the field of current medicinal and pharmacological research; and reported to have a broad spectrum of biological activities, such as anti-inflammatory (Da Sliva *et al.*, 2010), antitumor (Farag *et al.*, 2010), analgesic (Khode *et al.*, 2009), antimicrobial (Boschi *et al.*, 2011), anticancer, radioprotective (Ghorab *et al.*, 2010), antiamoebic (Husain *et al.*, 2008), antioxidant (Taj *et al.*, 2011) and antihypertensive (Mikhaylichenko *et al.*, 2009). In addition, pyrazoles have gained prominent role in developing the theory of heterocyclic chemistry. With this potential and diverse background of pyrazole derivatives, we have synthesized the title compound to study its crystal structure.

In the molecule of the title compound (Fig. 1), the dihedral angles between the benzene at the N-(C10—C15) and α- position (C16—C21) of the pyrazole ring (C1/N1/N2/C4/C5) are 8.52 (13) and 9.26 (12)°, respectively. The dihedral angle between the two benzene rings is 1.86 (13)°. The central pyrazole moiety adopts a highly flattened envelope conformation with puckering parameter Q = 0.128 (2) Å and φ = 325.4 (10)° (Cremer & Pople, 1975), and the maximum deviation found on the puckered atom at C1 is 0.078 (8) Å. The carboximidamide unit is in *syn-clinal* conformation with respect to the pyrazole moiety, as indicated by the torsion angle value of 78.0 (2)°. Bond lengths (Allen *et al.*, 1987) and bond angles agree with the observed values and are comparable to a related structure (Fun *et al.*, 2011). The molecules are linked into centrosymetric dimers *via* O9–H9···N8 hydrogen bonds (Table 1) and further weak N—H···N interactions make these centrosymmetric dimer to form one-dimensional chain. The molecular packing exhibits layered stacking when viewd down the 'a' axis as shown in Fig. 2.

### Experimental

A mixture of 1,3-diphenyl-4,5-dihydro-1*H*-pyrazole-5-carbonitrile (1.0 g,4.04 mmol), NH_2_OH.HCl (0.3 g, 4.04 mmol) and sodium carbonate (0.43 g, 4.04 mmol) in 50% ethanol and water (20 ml) was warmed on a water bath for 4–5 h. The progress of the reaction was monitored by TLC. After completion of the reaction the solvent was evaporated in vacuum. Then the reaction mass was quenched into crushed ice and left over night. The solid obtained was filtered, washed with water, dried and recrystallized from ethanol(m.p=204–206°C).

### Refinement

H atoms were placed at idealized positions and allowed to ride on their parent atoms with C–H distances in the range of 0.93 to 0.98 Å, and N–H distance of 0.86 Å; *U*_iso_(H) = 1.2*U*_eq_(carrier atom) for all H atoms.

### Crystal data

**Table:**

C_16_H_16_N_4_O	*Z* = 2
*M**_r_* = 280.33	*F*(000) = 296
Triclinic, *P*1	*D*_x_ = 1.299 Mg m^−^^3^
Hall symbol: -P 1	Melting point: 481 K
*a* = 7.845 (11) Å	Mo *K*α radiation, λ = 0.71073 Å
*b* = 8.940 (12) Å	Cell parameters from 2480 reflections
*c* = 11.116 (15) Å	θ = 1.9–25.0°
α = 99.50 (2)°	µ = 0.09 mm^−^^1^
β = 99.76 (2)°	*T* = 293 K
γ = 106.70 (2)°	Block, yellow
*V* = 716.8 (17) Å^3^	0.30 × 0.22 × 0.15 mm

### Data collection

**Table:**

Bruker APEXII CCD area-detector diffractometer	*R*_int_ = 0.031
ω and φ scans	θ_max_ = 25.0°, θ_min_ = 1.9°
6110 measured reflections	*h* = −9→9
2480 independent reflections	*k* = −10→10
1912 reflections with *I* > 2σ(*I*)	*l* = −13→13

### Refinement

**Table:**

Refinement on *F*^2^	Secondary atom site location: difference Fourier map
Least-squares matrix: full	Hydrogen site location: inferred from neighbouring sites
*R*[*F*^2^ > 2σ(*F*^2^)] = 0.053	H-atom parameters constrained
*wR*(*F*^2^) = 0.172	*w* = 1/[σ^2^(*F*_o_^2^) + (0.1095*P*)^2^ + 0.0579*P*] where *P* = (*F*_o_^2^ + 2*F*_c_^2^)/3
*S* = 1.09	(Δ/σ)_max_ = 0.001
2480 reflections	Δρ_max_ = 0.22 e Å^−^^3^
192 parameters	Δρ_min_ = −0.29 e Å^−^^3^
0 restraints	Extinction correction: *SHELXL97* (Sheldrick, 2008), FC^*^=KFC[1+0.001XFC^2^Λ^3^/SIN(2Θ)]^-1/4^
Primary atom site location: structure-invariant direct methods	Extinction coefficient: 0.039 (10)

### Special details

**Table:**

Geometry. Bond distances, angles *etc*. have been calculated using the rounded fractional coordinates. All su's are estimated from the variances of the (full) variance-covariance matrix. The cell e.s.d.'s are taken into account in the estimation of distances, angles and torsion angles
Refinement. Refinement on *F*^2^ for ALL reflections except those flagged by the user for potential systematic errors. Weighted *R*-factors *wR* and all goodnesses of fit *S* are based on *F*^2^, conventional *R*-factors *R* are based on *F*, with *F* set to zero for negative *F*^2^. The observed criterion of *F*^2^ > σ(*F*^2^) is used only for calculating -*R*-factor-obs *etc*. and is not relevant to the choice of reflections for refinement. *R*-factors based on *F*^2^ are statistically about twice as large as those based on *F*, and *R*-factors based on ALL data will be even larger.

### Fractional atomic coordinates and isotropic or equivalent isotropic displacement parameters (Å^2^)

**Table:**

	*x*	*y*	*z*	*U*_iso_*/*U*_eq_	
O9	−0.5121 (2)	0.68205 (19)	−0.45801 (14)	0.0562 (5)	
N2	0.0152 (2)	0.8338 (2)	−0.12879 (14)	0.0424 (5)	
N3	0.0582 (2)	0.81960 (19)	−0.00444 (14)	0.0404 (5)	
N7	−0.3248 (3)	0.8676 (2)	−0.24261 (16)	0.0516 (6)	
N8	−0.3688 (2)	0.6340 (2)	−0.39332 (14)	0.0444 (5)	
C1	−0.1393 (3)	0.6924 (2)	−0.20673 (17)	0.0396 (6)	
C4	−0.0671 (3)	0.6971 (2)	0.01121 (17)	0.0383 (6)	
C5	−0.2139 (3)	0.6106 (3)	−0.10645 (18)	0.0447 (6)	
C6	−0.2839 (3)	0.7360 (2)	−0.28863 (16)	0.0376 (6)	
C10	0.1498 (3)	0.9301 (2)	−0.17755 (17)	0.0386 (6)	
C11	0.3162 (3)	1.0346 (3)	−0.09989 (18)	0.0443 (6)	
C12	0.4414 (3)	1.1360 (3)	−0.1493 (2)	0.0549 (8)	
C13	0.4073 (3)	1.1382 (3)	−0.2751 (2)	0.0559 (8)	
C14	0.2440 (3)	1.0334 (3)	−0.3529 (2)	0.0570 (8)	
C15	0.1173 (3)	0.9290 (3)	−0.30604 (19)	0.0531 (7)	
C16	−0.0716 (3)	0.6542 (2)	0.13341 (18)	0.0405 (6)	
C17	0.0704 (3)	0.7310 (3)	0.24058 (19)	0.0503 (7)	
C18	0.0566 (4)	0.6911 (3)	0.3551 (2)	0.0619 (9)	
C19	−0.1001 (4)	0.5748 (4)	0.3648 (2)	0.0660 (10)	
C20	−0.2370 (4)	0.4957 (4)	0.2606 (3)	0.0725 (10)	
C21	−0.2240 (3)	0.5340 (3)	0.1455 (2)	0.0592 (8)	
H1	−0.09220	0.62150	−0.25880	0.0480*	
H5A	−0.32990	0.62440	−0.09850	0.0540*	
H5B	−0.22930	0.49700	−0.12590	0.0540*	
H7A	−0.41430	0.88920	−0.28410	0.0620*	
H7B	−0.26140	0.92980	−0.17180	0.0620*	
H9	−0.57860	0.60770	−0.51580	0.0840*	
H11	0.34250	1.03590	−0.01490	0.0530*	
H12	0.55100	1.20410	−0.09660	0.0660*	
H13	0.49130	1.20810	−0.30680	0.0670*	
H14	0.21920	1.03300	−0.43780	0.0680*	
H15	0.01040	0.85810	−0.36010	0.0640*	
H17	0.17460	0.80920	0.23490	0.0600*	
H18	0.15190	0.74200	0.42530	0.0740*	
H19	−0.11120	0.55120	0.44210	0.0790*	
H20	−0.33940	0.41570	0.26670	0.0870*	
H21	−0.31800	0.47900	0.07530	0.0710*	

### Atomic displacement parameters (Å^2^)

**Table:**

	*U*^11^	*U*^22^	*U*^33^	*U*^12^	*U*^13^	*U*^23^
O9	0.0510 (10)	0.0600 (10)	0.0460 (8)	0.0137 (8)	−0.0075 (7)	0.0075 (7)
N2	0.0361 (9)	0.0490 (10)	0.0306 (8)	0.0011 (8)	0.0024 (7)	0.0059 (7)
N3	0.0362 (9)	0.0463 (10)	0.0330 (8)	0.0083 (8)	0.0051 (7)	0.0059 (7)
N7	0.0511 (11)	0.0484 (11)	0.0476 (10)	0.0179 (9)	−0.0011 (8)	−0.0001 (8)
N8	0.0403 (10)	0.0487 (10)	0.0347 (8)	0.0086 (8)	−0.0013 (7)	0.0042 (7)
C1	0.0330 (11)	0.0389 (11)	0.0370 (10)	0.0049 (8)	0.0030 (8)	−0.0012 (8)
C4	0.0315 (10)	0.0405 (11)	0.0400 (10)	0.0096 (8)	0.0065 (8)	0.0070 (8)
C5	0.0380 (11)	0.0414 (11)	0.0458 (11)	0.0049 (9)	0.0019 (9)	0.0084 (9)
C6	0.0333 (10)	0.0372 (10)	0.0351 (9)	0.0037 (8)	0.0074 (8)	0.0034 (8)
C10	0.0338 (11)	0.0416 (11)	0.0392 (10)	0.0114 (9)	0.0088 (8)	0.0072 (8)
C11	0.0379 (11)	0.0495 (12)	0.0384 (10)	0.0070 (9)	0.0056 (9)	0.0072 (9)
C12	0.0411 (12)	0.0530 (14)	0.0594 (13)	0.0007 (10)	0.0082 (10)	0.0116 (11)
C13	0.0506 (14)	0.0582 (14)	0.0635 (14)	0.0130 (11)	0.0223 (11)	0.0254 (11)
C14	0.0554 (14)	0.0746 (16)	0.0449 (12)	0.0188 (12)	0.0157 (10)	0.0237 (11)
C15	0.0420 (12)	0.0675 (15)	0.0394 (11)	0.0071 (11)	0.0041 (9)	0.0082 (10)
C16	0.0393 (11)	0.0427 (11)	0.0425 (11)	0.0169 (9)	0.0105 (9)	0.0104 (9)
C17	0.0548 (14)	0.0448 (12)	0.0452 (11)	0.0096 (10)	0.0089 (10)	0.0083 (9)
C18	0.0827 (18)	0.0666 (16)	0.0392 (12)	0.0324 (14)	0.0080 (12)	0.0109 (11)
C19	0.0746 (19)	0.092 (2)	0.0582 (14)	0.0453 (17)	0.0318 (14)	0.0400 (14)
C20	0.0482 (15)	0.100 (2)	0.0864 (19)	0.0244 (15)	0.0253 (14)	0.0567 (17)
C21	0.0396 (13)	0.0733 (16)	0.0640 (14)	0.0105 (11)	0.0085 (11)	0.0311 (12)

### Geometric parameters (Å, º)

**Table:**

O9—N8	1.439 (3)	C16—C21	1.402 (4)
O9—H9	0.8200	C16—C17	1.404 (4)
N2—C1	1.490 (3)	C17—C18	1.391 (4)
N2—C10	1.406 (3)	C18—C19	1.397 (5)
N2—N3	1.402 (3)	C19—C20	1.366 (5)
N3—C4	1.303 (3)	C20—C21	1.391 (4)
N7—C6	1.350 (3)	C1—H1	0.9800
N8—C6	1.291 (3)	C5—H5A	0.9700
N7—H7B	0.8600	C5—H5B	0.9700
N7—H7A	0.8600	C11—H11	0.9300
C1—C5	1.538 (4)	C12—H12	0.9300
C1—C6	1.513 (4)	C13—H13	0.9300
C4—C16	1.474 (3)	C14—H14	0.9300
C4—C5	1.510 (4)	C15—H15	0.9300
C10—C15	1.406 (3)	C17—H17	0.9300
C10—C11	1.405 (4)	C18—H18	0.9300
C11—C12	1.387 (4)	C19—H19	0.9300
C12—C13	1.383 (4)	C20—H20	0.9300
C13—C14	1.391 (4)	C21—H21	0.9300
C14—C15	1.390 (4)		
			
O9···N7	2.596 (4)	H1···H15	2.5500
O9···N8^i^	2.829 (5)	H1···C18^vii^	2.9300
O9···H7A	2.2900	H1···C19^vii^	2.8600
O9···H9^i^	2.8600	H1···C20^vii^	3.0600
O9···H20^ii^	2.7400	H5A···N7	2.9000
N2···N7	2.873 (5)	H5A···C21	3.0100
N7···O9	2.596 (4)	H5A···H21	2.5000
N7···N2	2.873 (5)	H5B···C21	2.9700
N8···N8^i^	3.039 (5)	H5B···H21	2.4700
N8···O9^i^	2.829 (5)	H5B···C16^vii^	3.0400
N2···H7B	2.5500	H5B···C17^vii^	2.9100
N3···H11	2.5300	H7A···O9	2.2900
N3···H17	2.6900	H7A···C12^ix^	3.0500
N3···H7B^iii^	2.6200	H7A···C13^ix^	2.9500
N7···H17^iii^	2.7700	H7B···N2	2.5500
N7···H5A	2.9000	H7B···N3^iii^	2.6200
N7···H11^iii^	2.8900	H7B···C11^iii^	3.1000
N8···H9^i^	2.1200	H7B···H11^iii^	2.2700
N8···H20^ii^	2.8500	H7B···H17^iii^	2.4900
C6···C15	3.181 (6)	H9···O9^i^	2.8600
C11···C11^iv^	3.597 (6)	H9···N8^i^	2.1200
C15···C6	3.181 (6)	H11···N3	2.5300
C1···H15	2.6400	H11···N7^iii^	2.8900
C5···H21	2.6300	H11···C11^iv^	3.0400
C6···H15	2.5700	H11···H7B^iii^	2.2700
C6···H20^ii^	2.9500	H15···C1	2.6400
C11···H7B^iii^	3.1000	H15···C6	2.5700
C11···H11^iv^	3.0400	H15···H1	2.5500
C12···H7A^v^	3.0500	H17···N3	2.6900
C13···H7A^v^	2.9500	H17···N7^iii^	2.7700
C14···H18^vi^	3.1000	H17···H7B^iii^	2.4900
C15···H1	2.9500	H18···C14^x^	3.1000
C16···H5B^vii^	3.0400	H19···C19^viii^	3.0500
C17···H5B^vii^	2.9100	H19···H19^viii^	2.4700
C18···H1^vii^	2.9300	H20···O9^ii^	2.7400
C19···H1^vii^	2.8600	H20···N8^ii^	2.8500
C19···H19^viii^	3.0500	H20···C6^ii^	2.9500
C20···H1^vii^	3.0600	H21···C5	2.6300
C21···H5A	3.0100	H21···H5A	2.5000
C21···H5B	2.9700	H21···H5B	2.4700
H1···C15	2.9500		
			
N8—O9—H9	109.00	C18—C19—C20	119.9 (2)
N3—N2—C10	119.54 (16)	C19—C20—C21	120.4 (3)
C1—N2—C10	123.69 (15)	C16—C21—C20	121.2 (2)
N3—N2—C1	111.45 (15)	N2—C1—H1	110.00
N2—N3—C4	108.91 (15)	C5—C1—H1	110.00
O9—N8—C6	110.57 (17)	C6—C1—H1	110.00
H7A—N7—H7B	120.00	C1—C5—H5A	111.00
C6—N7—H7A	120.00	C1—C5—H5B	111.00
C6—N7—H7B	120.00	C4—C5—H5A	111.00
N2—C1—C6	113.72 (15)	C4—C5—H5B	111.00
C5—C1—C6	111.87 (19)	H5A—C5—H5B	109.00
N2—C1—C5	101.89 (15)	C10—C11—H11	120.00
N3—C4—C5	113.12 (17)	C12—C11—H11	120.00
N3—C4—C16	123.32 (18)	C11—C12—H12	119.00
C5—C4—C16	123.43 (19)	C13—C12—H12	119.00
C1—C5—C4	102.90 (18)	C12—C13—H13	121.00
N7—C6—C1	118.32 (16)	C14—C13—H13	121.00
N8—C6—C1	115.74 (17)	C13—C14—H14	119.00
N7—C6—N8	125.7 (2)	C15—C14—H14	119.00
N2—C10—C11	121.61 (17)	C10—C15—H15	120.00
N2—C10—C15	120.30 (19)	C14—C15—H15	120.00
C11—C10—C15	118.0 (2)	C16—C17—H17	120.00
C10—C11—C12	120.36 (18)	C18—C17—H17	120.00
C11—C12—C13	121.6 (2)	C17—C18—H18	120.00
C12—C13—C14	118.3 (2)	C19—C18—H18	120.00
C13—C14—C15	121.2 (2)	C18—C19—H19	120.00
C10—C15—C14	120.4 (2)	C20—C19—H19	120.00
C4—C16—C17	122.66 (19)	C19—C20—H20	120.00
C4—C16—C21	119.56 (19)	C21—C20—H20	120.00
C17—C16—C21	117.78 (19)	C16—C21—H21	119.00
C16—C17—C18	120.6 (2)	C20—C21—H21	119.00
C17—C18—C19	120.1 (2)		
			
C1—N2—N3—C4	−9.1 (2)	C16—C4—C5—C1	−176.4 (2)
C10—N2—N3—C4	−164.28 (18)	N3—C4—C16—C17	−7.4 (3)
N3—N2—C1—C5	13.1 (2)	N3—C4—C16—C21	171.5 (2)
N3—N2—C1—C6	133.67 (17)	C5—C4—C16—C17	176.9 (2)
C10—N2—C1—C5	167.10 (19)	C5—C4—C16—C21	−4.2 (3)
C10—N2—C1—C6	−72.4 (3)	N2—C10—C11—C12	−175.7 (2)
N3—N2—C10—C11	−11.3 (3)	C15—C10—C11—C12	1.6 (4)
N3—N2—C10—C15	171.45 (19)	N2—C10—C15—C14	174.8 (2)
C1—N2—C10—C11	−163.3 (2)	C11—C10—C15—C14	−2.5 (4)
C1—N2—C10—C15	19.4 (3)	C10—C11—C12—C13	0.2 (4)
N2—N3—C4—C5	0.5 (2)	C11—C12—C13—C14	−1.1 (4)
N2—N3—C4—C16	−175.53 (19)	C12—C13—C14—C15	0.2 (4)
O9—N8—C6—N7	1.8 (3)	C13—C14—C15—C10	1.6 (4)
O9—N8—C6—C1	175.42 (16)	C4—C16—C17—C18	177.3 (2)
N2—C1—C5—C4	−11.6 (2)	C21—C16—C17—C18	−1.7 (4)
C6—C1—C5—C4	−133.48 (17)	C4—C16—C21—C20	−176.9 (2)
N2—C1—C6—N7	−36.7 (3)	C17—C16—C21—C20	2.1 (4)
N2—C1—C6—N8	149.19 (18)	C16—C17—C18—C19	−0.6 (4)
C5—C1—C6—N7	78.0 (2)	C17—C18—C19—C20	2.5 (5)
C5—C1—C6—N8	−96.1 (2)	C18—C19—C20—C21	−2.1 (5)
N3—C4—C5—C1	7.6 (3)	C19—C20—C21—C16	−0.2 (5)

Symmetry codes: (i) −*x*−1, −*y*+1, −*z*−1; (ii) −*x*−1, −*y*+1, −*z*; (iii) −*x*, −*y*+2, −*z*; (iv) −*x*+1, −*y*+2, −*z*; (v) *x*+1, *y*, *z*; (vi) *x*, *y*, *z*−1; (vii) −*x*, −*y*+1, −*z*; (viii) −*x*, −*y*+1, −*z*+1; (ix) *x*−1, *y*, *z*; (x) *x*, *y*, *z*+1.

### Hydrogen-bond geometry (Å, º)

**Table:**

*D*—H···*A*	*D*—H	H···*A*	*D*···*A*	*D*—H···*A*
N7—H7*A*···O9	0.86	2.29	2.596 (4)	101
N7—H7*B*···N2	0.86	2.55	2.873 (5)	103
N7—H7*B*···N3^iii^	0.86	2.62	3.449 (5)	164
O9—H9···N8^i^	0.82	2.12	2.829 (5)	145
C11—H11···N3	0.93	2.53	2.843 (5)	100

Symmetry codes: (i) −*x*−1, −*y*+1, −*z*−1; (iii) −*x*, −*y*+2, −*z*.
